# The influence of age and health status for outcomes after mid-urethral sling surgery—a nationwide register study

**DOI:** 10.1007/s00192-022-05364-6

**Published:** 2022-10-01

**Authors:** Julia Gyhagen, Sigvard Åkervall, Jennie Larsudd-Kåverud, Mattias Molin, Ian Milsom, Adrian Wagg, Maria Gyhagen

**Affiliations:** 1 Department of Geriatrics, Dalens Hospital, Stockholm, Sweden; 2 Gothenburg Continence Research Centre, Institute of Clinical Sciences, Sahlgrenska Academy at Gothenburg University, Gothenburg, Sweden; 3 Department of Obstetrics and Gynecology, Södra Älvsborgs Hospital, SE–501 82 Borås, Sweden; 4 Statistical Consultancy Group, Gothenburg, Sweden; 5 Department of Obstetrics and Gynecology, Sahlgrenska University Hospital, Gothenburg, Sweden; 6 Division of Geriatric Medicine, University of Alberta, Edmonton, Alberta Canada

**Keywords:** ASA class, De novo urgency, Mixed urinary incontinence, Old age, Remission, Stress urinary incontinence, Urgency incontinence

## Abstract

**Introduction and hypothesis:**

The efficacy of mid-urethral sling (MUS) surgery in older women and women with a significant disease burden is limited. We aimed to determine the influence of chronological age and physical status (assessed by the American Society of Anesthesiologists Physical Status, ASA) classification on outcomes.

**Methods:**

Cure rate, change in frequency of lower urinary tract symptoms, satisfaction, impact, and adverse events after MUS surgery were assessed in 5200 women aged 55–94 years with MUS surgery (2010–2017). Data were analysed by multivariate logistic regression and Mantel-Haenszel chi-square statistics.

**Results:**

The cure rate was 64.2% (95% CI, 60.0–68.4) in the ≥ 75-year cohort compared to 88.5% (95% CI, 87.1–89.8) in the 55–64-year cohort (trend *p* < 0.0001). The estimated probability of cure, improvement, and satisfaction with the procedure decreased by aOR_10yr_ = 0.51 for cure to aOR_10yr_ = 0.59 for satisfaction (all *p* < 0.0001). Women with a significant health burden (ASA class 3–4) had lower cure rates and satisfaction than those without (65.5% vs. 83.7%, *p* < 0.0001 and 65.7% vs. 80.6%, *p* < 0.0001). Older age was more likely to be associated with de novo urgency (*p* = 0.0022) and nocturia ≥ 2 (*p* < 0.0001). Adverse events, readmission, and 30-day mortality rates were low. Women, irrespective of age, were equally satisfied if they experienced a decrease of at least one step in leakage frequency.

**Conclusions:**

Even if MUS surgery in older women and those with ASA class 3–4 was associated with a lower cure rate and less satisfactory outcome, a majority were satisfied provided they experienced a reduction of incontinence episodes.

**Supplementary Information:**

The online version contains supplementary material available at 10.1007/s00192-022-05364-6.

## Introduction

Stress urinary incontinence (SUI) [1] affects 25–45% of older women and incurs a high cost for the individual and society [2, 3]. The mid-urethral sling (MUS) is a highly effective, safe, and less invasive procedure than prior surgical techniques for SUI [4–6]. Many factors [comorbidity, persistent urgency urinary incontinence (UUI), and intrinsic sphincter deficiency (ISD)] are known to contribute to less favourable outcomes in older women [7, 8]. In the UK, the number of these procedures in older women decreased between 2000–2012 [9] and these procedures are now prohibited (https://www.immdsreview.org.uk/Report.html), in contrast to numerous European countries. MUS procedures are still being performed in the Nordic countries, where they continue to be the evidence-based gold standard. We hypothesised that physical status might be important for the outcome of surgery. The aim of this study was to evaluate the influence of chronological age and ASA class (The American Society of Anesthesiologists Physical Status classification system) on the efficacy of the procedure and adverse events at a 1-year follow-up [10].

## Materials and methods

This study was a national register-based cohort study. Information was extracted from the Swedish National Quality Register of Gynecological Surgery (GynOp). GynOp was started in 1997 and was intended for audit and research purposes. The County Council of Västerbotten, Umeå, Sweden, is the legal and responsible owner of the register. The section about SUI surgery has been in use since 2006. The objective of GynOp is to systematically describe, report, develop, and ensure the quality of women's health care. GynOp consists of six independent and cooperating registries and covers all major surgical procedures in gynaecology (https://www.gynop.se/home). Coverage in the continence surgery section was > 90% in 2017. Information was prospectively and consecutively collected throughout the health care process, based on a preoperative evaluation (with postal- or web-based questionnaires), hospital records from admission, surgery, discharge, and a postoperative questionnaire. All women planned for surgery received written information about GynOp and were informed about the possibility of not participating or opting out at any time. The Swedish Association of Local Authorities and Regions has reviewed and certified that the pre- and postoperative questionnaire (8 weeks and 12 months) concerning patient reported outcomes has good face and content validity, and it was assigned the highest degree of certification (level 1) [11]. Follow-up of surgical results on MUS with self-report has been shown to be effective and corresponds well with objective findings [12].

### Study population

All women with SUI, with or without UUI, aged ≥ 55 years, who had MUS surgery (both transvaginal retropubic and transobturator procedures) from 2010 to 2017 were eligible for the study (*n* = 5200). Surgery was performed as a day case or inpatient case under local, regional, or general anaesthesia. Women with prior continence surgery were included, while those with concomitant prolapse surgery were excluded. The women were stratified into three age cohorts: 55–64, 65–74, and 75–94 years.

### The questionnaires

The preoperative questionnaire included height, weight, parity, prior abdominal and gynaecological surgery, co-existing medical conditions, and physical performance. The questionnaires consisted of validated questions about lower urinary tract symptoms (LUTS) [13]. An anaesthesiologist performed an ASA classification at the time of the intervention. Women in ASA class 1–2 (healthy or mild systemic disease) were categorised as healthy, and those with ASA class 3–4 (severe systemic disease with and without a constant threat to life) were classified as having a significant disease burden. SUI was defined by the question “How often do you experience leakage of urine associated with physical activity, or when you laugh, cough, or sneeze?” followed by the options “Never”, “1-4 times per month”, (no SUI) and “1-6 times per week”, “once a day”, and “more than once a day” (SUI). UUI was defined by the question “How often do you experience a sudden onset of a strong need to urinate and leak urine before you reach the toilet?” followed by the options “Never”, “1-4 times per month” (= no UUI), “1-6 times per week”, “once a day”, and “more than once a day” (= UUI). MUI was defined as having SUI and UUI in combination [4]. Urinary urgency was evaluated by the question “Have you had problems with a sudden onset of a strong need to urinate?” and deemed positive by the answers “1–3 times/week” or more often. Nocturia was defined by usually urinating ≥ 2 times/night. Women were also asked about difficulty emptying the bladder. A positive response was defined as difficulties occurring 1–3 times/week or more often. Body mass index (BMI, kg/m^2^) was calculated using information from the preoperative questionnaire. The preoperative questionnaire is available at https://www.gynop.se/wp-content/uploads/2019/01/Questionnaire-prior-to-surgery-190115.pdf.

Postoperatively, cure was defined as SUI “Never” or “1-4 times per month”. The patients' satisfaction with the operation was grouped into satisfied (very satisfied and satisfied) versus dissatisfied (neither satisfied nor dissatisfied, dissatisfied, and very dissatisfied). Improvement of SUI was defined by the answers greatly improved and improved versus unchanged or worse. Failure was defined as unchanged and worse, i.e., the same or a higher frequency of leakage postoperatively. Women confirmed de novo symptoms of urgency and difficulty with emptying the bladder by reporting the frequency of < 3 times/month preoperatively versus > 1–3 times/week or more often postoperatively. De novo nocturia denoted a change from < 2/night to ≥ 2 times/night. Remission was defined as having the symptom ≥ 1/week preoperatively versus < 1/week 1-year postoperatively. The postoperative questionnaire is available at https://www.gynop.se/wp-content/uploads/2019/01/Questionnaire-1-year-after-surgery-190115.pdf.

### Statistical analysis

Continuous variables are presented as mean and standard deviation and median and interquartile ranges. Categorical data are presented as number, percent, and 95% confidence interval (CI). Fisher’s exact test was used for dichotomous variables and Mann-Whitney *U*-test for continuous variables when comparing cohort characteristics between two groups. The calculation of the 95% CI for the difference in percentages between categorical variables was based on the exact method. Logistic regression models were used to calculate the age-related estimated probability of cure, improvement, and satisfaction of SUI per 10 years, adjusted for BMI and UUI. The trend was analysed with Mantel-Haenszel chi-square statistics. In each analysis, missing data were accounted for and excluded. No adjustment was made for multiple testing. All statistical testing was two sided, and the significance level was set to *p*< 0.05. Analyses were performed using SAS, version 9.4 (SAS Institute, Inc., Cary, NC).

## Results

### Demographics and preoperative status

According to GynOp, the proportion of women aged ≥ 75 years receiving MUS surgery more than halved from 8.9% in 2007 to 3.8% in 2017 (Trend *p* < 0.0001, Supplementary material, Fig. S1A). Across the same calendar period in the general population, the proportion of those aged ≥ 75 years was ~14% (Supplementary material, Fig. S1B). Preoperative rates of LUTS, ASA class 3–4, BMI ≥ 30, and prior surgery for SUI and pelvic organ prolapse (POP) increased with age (trend all *p* < 0.0001). The use of obturator slings was similar in each of the three age groups (~30%, *p* = 0.23) (Table 1).

**Table 1:** Preoperative cohort characteristics

	55–64 *N* = 2585	65–74 years *N* = 1949	≥ 75 years *N* = 666	Trend *p* value^a^
	Mean (SD) Median (IQR) (95% CI for mean)^b^			
Age, years	59.3 (2.9) 59 (57–62) (59.2–59.4)	69.0 (2.8) 69 (67–71) (68.9–69.1)	78.9 (3.5) 78 (76–81) (78.6–79.2)	
BMI (kg/m^2^)	26.8 (4.2) 26.2 (23.8–29.4) (26.7–27.0)	27.3 (4.5) 26.8 (24.1–30.1) (27.1–27.5)	27.3 (4.3) 26.7 (24.3–30.1) (27.0–27.6)	0.0005
	n/N % (95% CI)	n/N % (95% CI)	n/N % (95% CI)	
BMI ≥ 30 (kg/m^2^)	509/249,5 20.4 (18.8–22.0)	484/186,7 25.9 (24.0–28.0)	161/62,0 26.0 (22.6–29.6)	< 0.0001
Vaginal parity ≥ 3	887/242,3 36.6 (34.7–38.6)	666/185,4 35.9 (33.7–38.2)	279/63,6 43.9 (40.1–47.8)	0.014
Smoking ≥ 1 cigarette/day	313/254,2 12.3 (11.1–13.7)	159/19,11 8.3 (7.1–9.7)	23/651 3.5 (2.3–5.3)	< 0.0001
Hysterectomy	445/222,8 20.0 (18.3–21.7)	483/179,4 26.9 (24.9–29.0)	145/64,7 22.4 (19.3–25.8)	< 0.0001
Estrogen^c^	1347/253,1 53.2 (51.3–55.2)	1069/188,0 56.9 (54.6–59.1)	331/62,0 53.4 (49.4–57.4)	0.26
Heart disease	182/25,26 7.2 (6.2–8.3)	281/189,3 14.8 (13.3–16.5)	164/63,8 25.7 (22.4–29.3)	< 0.0001
Hypertension	813/249,9 32.5 (30.7–34.4)	917/188,9 48.5 (46.3–50.8)	356/63,9 55.7 (51.8–59.6)	< 0.0001
Diabetes	160/24,72 6.5 (5.5–7.5)	200/182,9 10.9 (9.5–12.5)	73/61,8 11.8 (9.4–14.6)	< 0.0001
Need to rest after two flights of stairs	227/25,34 9.0 (7.9–10.1)	365/189,2 19.3 (17.5–21.1)	215/63,1 34.1 (30.4–37.9)	< 0.0001
Need to rest after half a flight of stairs	37/15,40 2.4 (1.7–3.3)	33/11,41 2.9 (2.0–4.0)	27/397 6.8 (4.5–9.7)	= 0.0001
ASA^d^ class 3–4	69/25,54 2.7 (2.1–3.4)	106/19,18 5.5 (4.6–6.7)	72/65,6 11.0 (8.7–13.6)	< 0.0001
Urgency ≥ 1/week	1368/253,2 54.0 (52.1–56.0)	1171/186,6 62.8 (60.5–65.0)	455/61,0 74.6 (70.9–78.0)	< 0.0001
UUI ≥ 1/week^e^	1414/244,2 57.9 (55.9–59.9)	1276/180,8 70.6 (68.4–72.7)	506/61,1 82.8 (79.6–85.7)	< 0.0001
Nocturia ≥ 2/night	529/255,2 20.7 (19.2–22.4)	633/191,8 33.0 (30.9–35.2)	351/65,5 53.6 (49.7–57.5)	< 0.0001
Difficulty emptying the bladder ≥ 1/week	362/250,8 14.4 (13.1–15.9)	301/184,2 16.3 (14.7–18.1)	127/59,1 21.5 (18.2–25.0)	< 0.0001
Prior UI surgery^f^	287/219,7 13.1 (11.7–14.5)	330/176,6 18.7 (16.9–20.6)	184/63,6 28.9 (25.4–32.6)	< 0.0001
Prior POP surgery^f^	113/21,90 5.2 (4.3–6.2)	174/17,51 9.9 (8.6–11.4)	82/63,6 12.9 (10.4–15.8)	< 0.0001
Transobturator sling	813/258,5 31.5 (29.7–33.3)	591/194,9 30.3 (28.3–32.4)	195/66,6 29.3 (25.9–32.9)	0.23

*SD*, standard deviation; *IQR*, interquartile range (Q1; Q3); *CI*, confidence interval; *BMI*, body mass index (kg/m^2^); *UI*, urinary incontinence; *POP*, pelvic organ prolapse; *UUI*, urgency UI. ^a^Trend was analysed with Mantel-Haenszel chi-square statistics. ^b^The calculation of the 95% confidence limits was based on the exact method. ^c^Both systemic and vaginal treatments. ^d^The American Society of Anesthesiologists Physical Status classification system. ^e^UUI in addition to SUI. ^f^Prior surgery as reported by the woman in the questionnaire

### Study population and 1-year outcomes

Five thousand two hundred women aged 55–94 years underwent MUS surgery from 2010–2017. A total of 4581 women (88%) answered the 1-year questionnaire: 55–64 years, *n* = 2252/2585 (87.1%), 65–74 years, *n* = 1754/1949 (90.0%), and ≥ 75 years, *n* = 575/666 (86.3%) (Table 2).

**Table 2:** Main outcomes 1 year after surgery according to age class

	55–64 years *N* = 2252	65–74 years *N* = 1754	≥ 75 years *N* = 575	Trend *p* value ^a^
	% (95% CI)^b^	% (95% CI)	% (95% CI)	
Cure^c^ SUI	1971/2228 88.5 (87.1–89.8)	1376/1692 81.3 (79.4–83.2)	336/52,3 64.2 (60.0–68.4)	< 0.0001
Cure^c^ UI	1449/1703 85.1 (83.3–86.7)	1197/1747 68.5 (66.3–70.7)	291/55,9 52.1 (47.8–56.3)	< 0.0001
Satisfaction	1903/2225 85.5 (84.0–87.0)	1342/1720 78.0 (76.0–80.0)	343/54,2 63.3 (59.1–67.4)	< 0.0001
Improvement	2034/2222 91.5 (90.3–92.7)	1449/1703 85.1 (83.3–86.7)	396/54,7 72.4 (68.4–76.1)	< 0.0001
*De novo* symptoms				
Urgency^d^	123/10,29 12.0 (10.0–14.1)	111/62,4 17.8 (14.9–21.0)	22/12,8 17.2 (11.1–24.9)	0.0022
Difficulty emptying the bladder	226/18,73 12.1 (10.6–13.6)	154/13,76 11.2 (9.6–13.0)	38/37,7 10.1 (7.2–13.6)	0.23
Nocturia ≥ 2/night	81/17,90 4.5 (3.6–5.6)	113/11,69 9.7 (8.0–11.5)	30/26,7 11.2 (7.7–15.7)	< 0.0001
Remission				
Urgency	664/11,70 56.8 (53.9–59.6)	537/10,43 51.5 (48.4–54.6)	152/36,3 41.9 (36.7–47.1)	< 0.0001
Difficulty emptying the bladder	181/30,0 60.3 (54.6–65.9)	156/26,4 59.1 (52.9–65.1)	61/98 62.2 (51.9–71.8)	0.87
Nocturia ≥ 2/night	216/43,4 49.8 (45.0–54.6)	228/56,1 40.6 (36.6–44.8)	80/28,4 28.2 (23.0–33.8)	< 0.0001
Complications				
Bladder injury	112/23,32 4.8 (4.0–5.8)	104/18,09 5.7 (4.7–6.9)	34/596 5.7 (4.0–7.9)	0.20
Ureteric injury	2/23,32 0.1 (0.0–0.3)	8/18,09 0.4 (0.2–0.9)	1/596 0.2 (0.0–0.9)	0.19
Fistulas	4/23,32 0.2 (0.1–0.4)	1/18,09 0.1 (0.0–0.3)	0/596 0.0 (0.0–0.0)	0.16
Readmission 30 days	38/25,85 1.5 (1.0–2.0)	17/19,49 0.9 (0.5–1.4)	2/666 0.3 (0.0–1.1)	0.0044
Mortality 30 days	1/25,85 0.0 (0.0–0.2)	0/19,49 0.0 (0.0–0.0)	0/666 0.0 (0.0–0.0)	0.37

Responders: 55–64 years, *n* = 2252/2585 (87.1%), 65–74 years, *n* = 1754/1949 (90.0%), ≥ 75 years, *n* = 575/666 (86.3%). CI, confidence interval; UI, urinary incontinence; SUI, stress UI. For categorical variables, n/N (%) and exact 95% CI are presented. ^a^Trend was analysed with Mantel-Haenszel chi-square statistics. ^b^The calculation of the 95% confidence limits was based on the exact method. ^c^Cure was defined as incontinence “Never” and “1-4 times per month”. ^d^ With and without incontinence

**Fig. 1:**
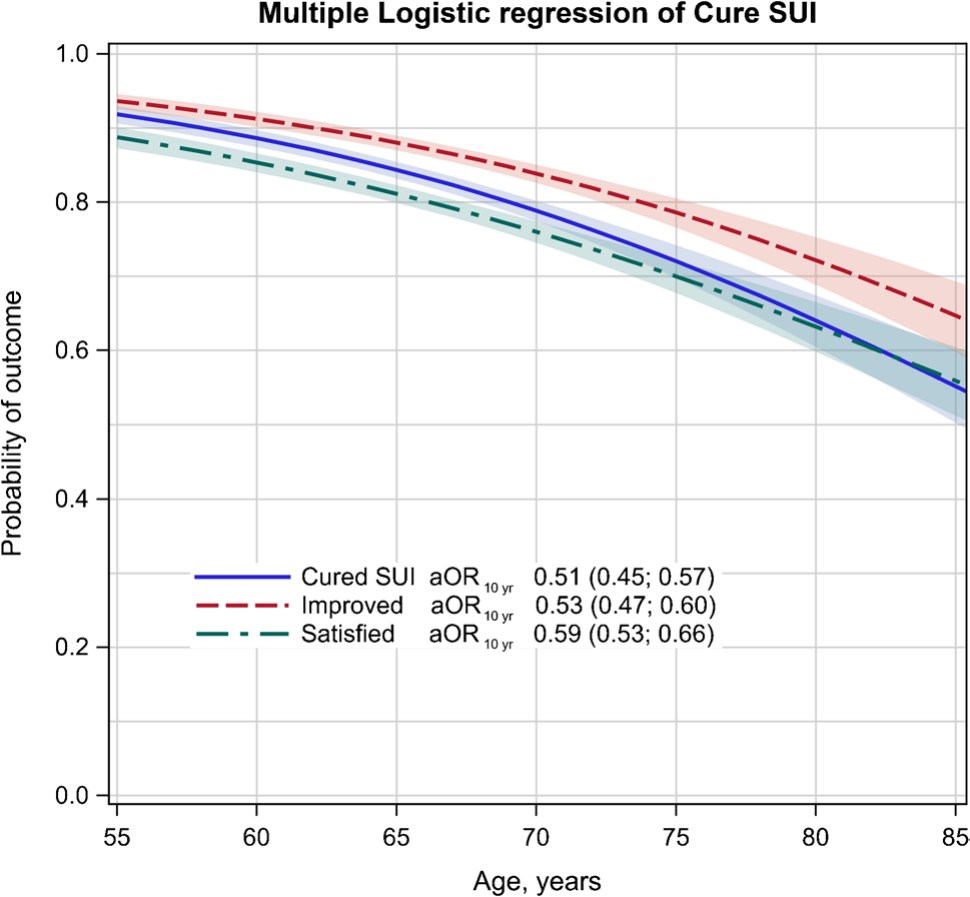
The probability of main outcomes according to age. SUI, stress urinary incontinence; aOR, adjusted OR. aOR was calculated from a logistic regression model with adjustment for body mass index (kg/m^2^) and urgency urinary incontinence

**Fig. 2:**
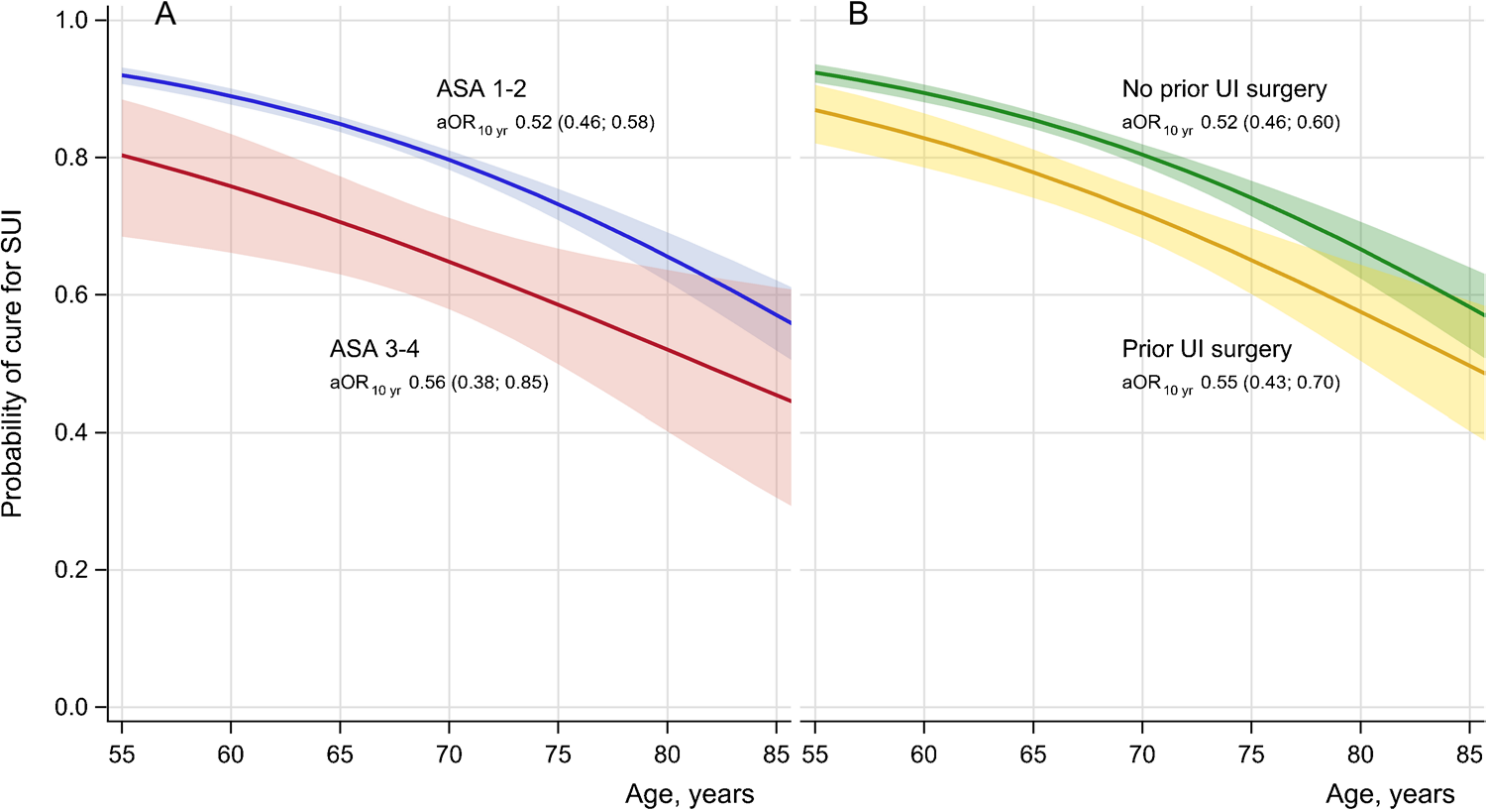
The probability of cure of stress urinary incontinence **A**. According to age and ASA class **B**. According to age and prior surgery. ASA is the classification system adopted by the American Society of Anesthesiologists; SUI, stress urinary incontinence; aOR, adjusted odds ratio. aOR was calculated from a logistic regression model with adjustment for body mass index (kg/m^2^) and urgency urinary incontinence. The estimated age-related probability (0–1) for the cure of SUI was calculated from logistic regression models, one for each risk factor. The shaded areas show the 95% two-tailed confidence intervals for the estimated probability of the mean. Prior surgery as reported in the questionnaire

The defined cure rate for SUI was 88.5% (95% CI, 87.1–89.8) in the 55–64 years cohort and 64.2% (95% CI, 60.0–68.4) in the ≥ 75 years cohort (trend *p* < 0.0001), decreasing by adjusted odds ratio per 10 years (aOR_10yr_), 0.51 [(95% CI, 0.45–0.57), *p* < 0.0001] (Table 2, Fig. 1). Lower rates for satisfaction [OR_10yr_, 0.59 (95% CI 0.53–0.66, *p* < 0.0001)] and improvement OR_10yr_, 0.53 (95% CI 0.47–0.60) were associated with increasing age (*p* < 0.0001) (Table 2, Fig. 1). The estimated age-related probability of cure of SUI, improvement, and satisfaction were similar across groups (Fig. 1). Change in frequency of leakage from the preoperative assessment to 1 year postoperatively is shown in Supplementary material, Fig. S2 A + B and Table S1 A + B. Of women ≥ 75 years, 76.1% experienced a reduction of leakage episodes compared with 92.0% in the cohort 55–74 years (*p* < 0.0001) (Supplementary material, Fig. S2 A + B and Table S2 A + B). Provided that there was a decrease of at least one step in leakage frequency, younger (55–74 years) and older women (≥ 75 years) were equally satisfied (Supplementary material, Fig. S2 A + B). Women with preoperative leakage, 1/week or less often, were less likely to achieve cure compared to women with more frequent leakage episodes (*p* < 0.0001) (Supplementary material, Fig. S3, and Table S2 A + B). Women with ASA class 3–4 were associated with a decreased cure rate of SUI compared to those with ASA class 1–2 (65.5% versus 83.7%, *p* < 0.0001) and a decreased rate of satisfaction (65.7% versus 80.6%) (both *p* < 0.0001) (Supplementary material, Table S3). The estimated probability across ages for cure was 10–15 percentage points lower for women in ASA class 3–4 and with prior UI surgery (Fig. 2A + B). There was a strong association between satisfaction and cure, regardless of ASA class and prior surgery (Supplementary material, Fig. 4 A + B). Cure for SUI and satisfaction were lower in women with diabetes (*p*<0.0001, not shown in Table). Prior surgery was associated with a decreased cure rate (84.3 versus 73.5%, *p* < 0.0001) and satisfaction (81.5 versus 71.2%, *p* < 0.0001, Supplementary material, Table S4 and Fig. S4 B).

### *De novo* symptoms, remission of symptoms, and complications

**Fig. 3:**
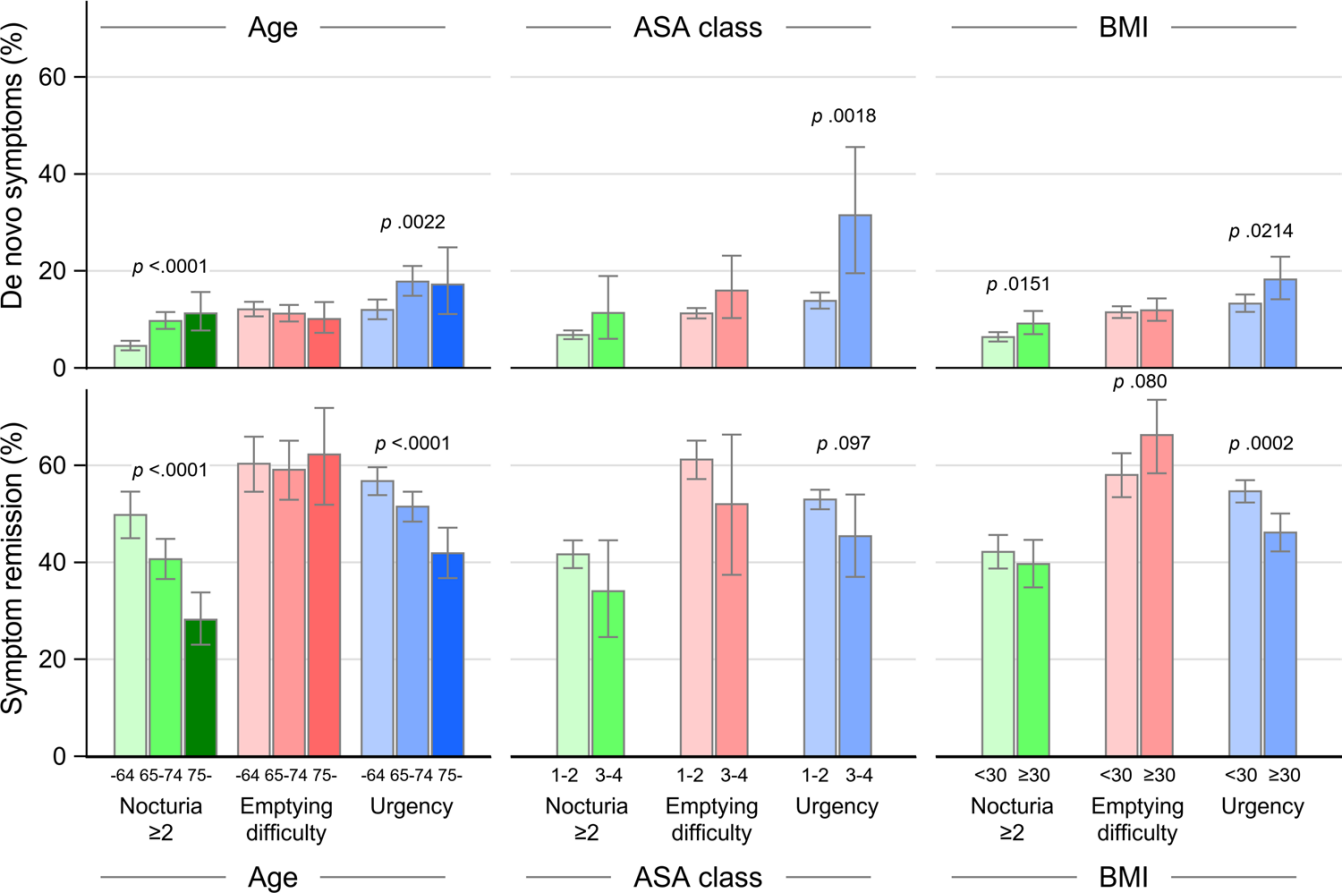
Remission and de novo lower urinary tract symptoms. ASA, the classification system adopted by the American Society of Anesthesiologists; BMI, body mass index (kg/m^2^); *p* values ≥ 0.10 are not shown

The rate of *de novo* urgency was lower in the 55–64-year age group compared with women aged ≥ 75 years (from 12.0% aged 55–64 years to 17.2% in those aged ≥ 75 years (trend *p* = 0.0022). De novo bladder emptying difficulty was similar across ages (trend *p* = 0.23) (Table 2, Fig. 3). Overall, de novo urgency was more common in ASA class 3–4 (*p* = 0.0018) (Fig. 3 and Supplementary material, Table S3). BMI ≥ 30 was associated with de novo urgency and de novo nocturia ≥ 2/night compared with BMI < 30 (*p* < 0.0002 and *p* <0.014) (Fig. 3). Prior surgery was also associated with increased rates of de novo urgency (23.9% versus 13.0%, *p* < 0.0001, Supplementary material, Table S4).

Remission of urgency and nocturia ≥ 2/night was common and most prevalent in the youngest age group (trend *p* < 0.0001) (Table 2, Fig. 3). ASA class did not affect remission of nocturia, difficulty with bladder emptying, and urgency (Fig. 3). Perioperative bladder perforations were similar in all age groups (4.8% to 5.7%, trend *p* = 0.20). Ureteric injury and fistulae were rare and similar regardless of age group (Table 2) and ASA class (Supplementary material, Table S3). The rate of readmission within 30 days was low (overall 1.1%, *n* = 55), and the mortality rate was 0.02% (*n* = 1, in the youngest group) (Table 2, Supplementary material, Table S3).

Ethical approvals for this study were obtained from the Regional Ethical Review Board in Gothenburg (reference no. 345-17; June 15, 2017) and Swedish Ethical Review Authority (reference no. 2020-01359; May 6, 2020). The study used an anonymised dataset, and all women gave their written consent to participate.

## Discussion

### Principal findings

The cure rate was 64.2% in the ≥ 75-year cohort compared to 88.5% in the 55–64-year cohort. Older age, ASA class 3–4, and prior surgery were associated with lower cure rates and less favourable outcomes. Furthermore, physical status and the presence of co-existing conditions seemed to be more important than chronological age for all outcomes. Women were satisfied with the procedure if they experienced a reduction in leakage episodes, irrespective of age. Overall measures of the result of MUS surgery (cure of SUI, improvement, and satisfaction with the procedure) seemed interchangeable. Women with a low preoperative rate of leakage were less likely to achieve our definition of cure. The rate of de novo urgency increased with age, and symptom remission was common in those with preoperative urgency and nocturia ≥ 2 and more pronounced in women aged 55–64 years. Overall, adverse events, readmissions, and 30-day mortality were rare in all age groups. The rate of MUS surgery in women aged ≥ 75 years more than halved in Sweden in 2007–2017.

### Results in context

Hellberg et al. [14] reported a cure rate of 62% after TVT surgery in 113 women aged ≥ 75 years, which is similar to the cure rate reported here (64%). In a Norwegian national register study from 2018 (*n* = 21,832), Engen et al. found a cure rate of 78.9% in women ≥ 70 years (6–12 months follow-up) [15]. In contrast, Stav et al. found no difference in subjective cure rate in 96 women (≥ 80 years) compared with 1016 women < 80 years of age (81 versus 85%, *p* = 0.32) with a mean follow-up of ~4 years [16]. The definition of cure differed between studies from “no objective leakage” [17] and “completely cured” [15] to “almost completely cured”/“cured”, and there is at present no consensus regarding the definition of cure, which varies according to study [6, 14, 17]. This study defined “no leakage” and “SUI less than once a week” as a cure. Most of the women in this study had urinary leakage > 1 per day preoperatively, and to be classified as cured, they had to have no leakage or leakage only 1–4 times per month postoperatively, which is clinically highly relevant. Stratification according to age differed: 75 years in our study and in that of Hellberg et al. [14]. Engen et al. used two groups, 70–79 years and 80–99 years, and Stav et al. set the cut-off at ≥ 80 years [15, 16]. Two studies [15, 16] used convenience samples from hospital centres, and two studies had a small number of older women (*n* = 113 and *n* = 96) [14, 15], which may explain why the results differed. This was further complicated by variable definitions of outcomes and age classes.

The rate of satisfaction in older women here was lower than that reported by Engen et al. [15] (70% and 63%). The higher rate in that study may be explained by the use of “Very satisfied” in a slightly younger cohort compared with the use of “Satisfied” plus “Very satisfied” here. Among the younger women here, the rate of “satisfaction” was almost the same (84% and 82%) with the same provisions. In this study, de novo urgency occurred in 12% (55–64 years) and 17% (≥ 75 years) (*p* =  0.0022) similar results have been reported by Hellberg et al. (14 vs. 21%, OR, 1.63; 95% CI 0.77–3.19) and Stav et al. (16% vs. 18%, *p* = 0.41) [14, 16]. Malek et al. reported lower rates (7.5% vs. 4.3%, *p* = 0.22), which may be explained by the definition of older women (≥ 70 years) [17]. We found a remission rate for urgency of ~55% in the younger age group, consistent with the study of Abdel-Fattah et al. on 83 women with a mean age of 55 years (56.1% at 1 year) [18]; however, this effect does not seem to persist over time [19]. We also observed a remission of symptoms of nocturia and bladder emptying difficulties in about every second woman, which may partly be attributed to the effect of surgery.

### Health status

At present, there is little information about the influence of physical status on the efficacy of MUS surgery, and we could not find any studies for comparison. For women with ASA class 3–4 in this study, results were less favourable regardless of age. In the study of Wai et al., women with diabetes had a lower satisfaction rate (*p* < 0.007) [20], and Stav et al. [16] found an increased risk of treatment failure 1 year postoperatively in this group (*p* < 0.05). We also found that the cure of SUI and satisfaction with the procedure were lower in women with diabetes (both *p* < 0.0001).

### Prior surgery

In this study, cure, satisfaction, and subjective improvement rates were ~10% lower in women with a history of prior surgery (all *p* < 0.0001). A recent systematic review on prior surgery for SUI showed a pooled success rate of 69% [21]. However, the 24 studies were heterogeneous regarding age, follow-up time, and the definition of “cured”. Prior surgery has also been linked to de novo urgency in three studies with 8% to 30% rates compared to our result (24%) [22–24]. Women with prior surgery have also been shown to be eight times more likely to use anticholinergic treatment for urgency after a MUS surgery (OR 8.2, 95% CI 1.3–13.3, *p* < 0.046) [25].

### Adverse events

We found a similar rate of bladder injury, fistulae, and ureteric injury regardless of age group and ASA class. Anger et al. reported no statistical difference in major surgical complications after MUS surgery between women aged 65–74 years (21.4%) versus women aged ≥ 75 years (21.3%) [26]. In contrast with our findings, a study of 7113 women from 2017 found that those with ASA class 3-4 had a higher 30-day readmission rate compared to women with ASA class 1-2 (2.3% versus 0.9%, *p* < 0.0001) [27], in contrast with our findings. Our results suggest that older women (≥ 75 years) and those with ASA class 3–4 should be informed preoperatively about the less favourable outcomes in most LUTS after MUS surgery. However, two out of three women were satisfied with the procedure's outcomes. Significant complications were rare and similar regardless of age group and ASA class.

### Strengths and limitations

The Swedish public health care system is similar to the National Health Service in the UK; medical assessment and surgery are available to all citizens, reducing the risk of systematic bias in reporting. The large cohorts were based on national registers, which offered prospective data with a response rate of almost 90%. The majority of women in this study were healthy, with no co-existing morbidities, which may have affected the external validity of the results. Most physicians did not physically see the participants postoperatively, which might be a limitation. However, several studies have shown that the occurrence and change in the severity over time of self-reported symptoms from a questionnaire are consistent and valid when they exist at the time of the report [28, 29]. Likewise, self-administered questionnaires are considered the most suitable tool for gathering information about sensitive issues [30]. A follow-up time of 1 year may also seem limited, but there is some evidence that most postoperative results remain stable beyond 1 year [14, 19]. We were unable to measure outcomes that older women might define as more relevant to them—functional/QoL and change in usual activities of daily living—as these data were not included in this primarily surgical dataset. In a study by Lo et al. on women with urodynamically proven SUI, it was shown that both the urodynamic and subjective cure rates decreased with age and that ISD was significantly associated with failure in older women [31]. Therefore, it might be a limitation that there was no information about ISD in the register: therefore, we could not assess the influence on the age-related outcomes here.

Understanding the outcome of MUS surgery in older women and women with comorbidities is vital as the proportion of such women in the population is increasing. We found a consistent declining trend for admission of women ≥ 55 years for MUS surgery. This is puzzling as the demand for surgery should have increased because of an increasing number of older women. There was no change in the annual capacity for MUS operations during the study period, so older women may simply have been given a lower priority [9]. A similar trend has also been documented in the UK, where the rate decreased from 7% to 5% between 2000–2011.

## Conclusion

Although the cure rate decreased with older age and higher ASA class, most women were satisfied if they experienced a reduction of incontinence episodes. Women with severe incontinence were more likely to be satisfied and improved. Our results highlight several important preoperative factors influencing the risk of failure such as chronological age and physical status, prior UI surgery, and diabetes. Although the results of this study demonstrate the ongoing downward trend in the number and share of MUS procedures in older women, they cannot explain them.

## Supplementary information

ESM 1 (DOCX 20324 kb)
