# Supplementary material for: The influence of age and health status for outcomes after mid-urethral sling surgery—a nationwide register study
Source: Int Urogynecol J. 2022 Oct 1;34(4):939–47. doi: 10.1007/s00192-022-05364-6 (PMC10038952; doi:10.1007/s00192-022-05364-6)
Supplement: Supplementary file 1 — (DOCX 20324 kb) [file 192_2022_5364_MOESM1_ESM.docx]

Supplementary material

The authors have provided this supplementary material to give readers additional information about their work. Supplement to: Gyhagen J, Åkervall S, Larsudd-Kåverud J, Molin M, Milsom I, Wagg A, Gyhagen M. The influence of age and health status for outcomes after

mid-urethral sling surgery – a nationwide register study

Figure S1A. Distribution of midurethral sling surgery according to age class


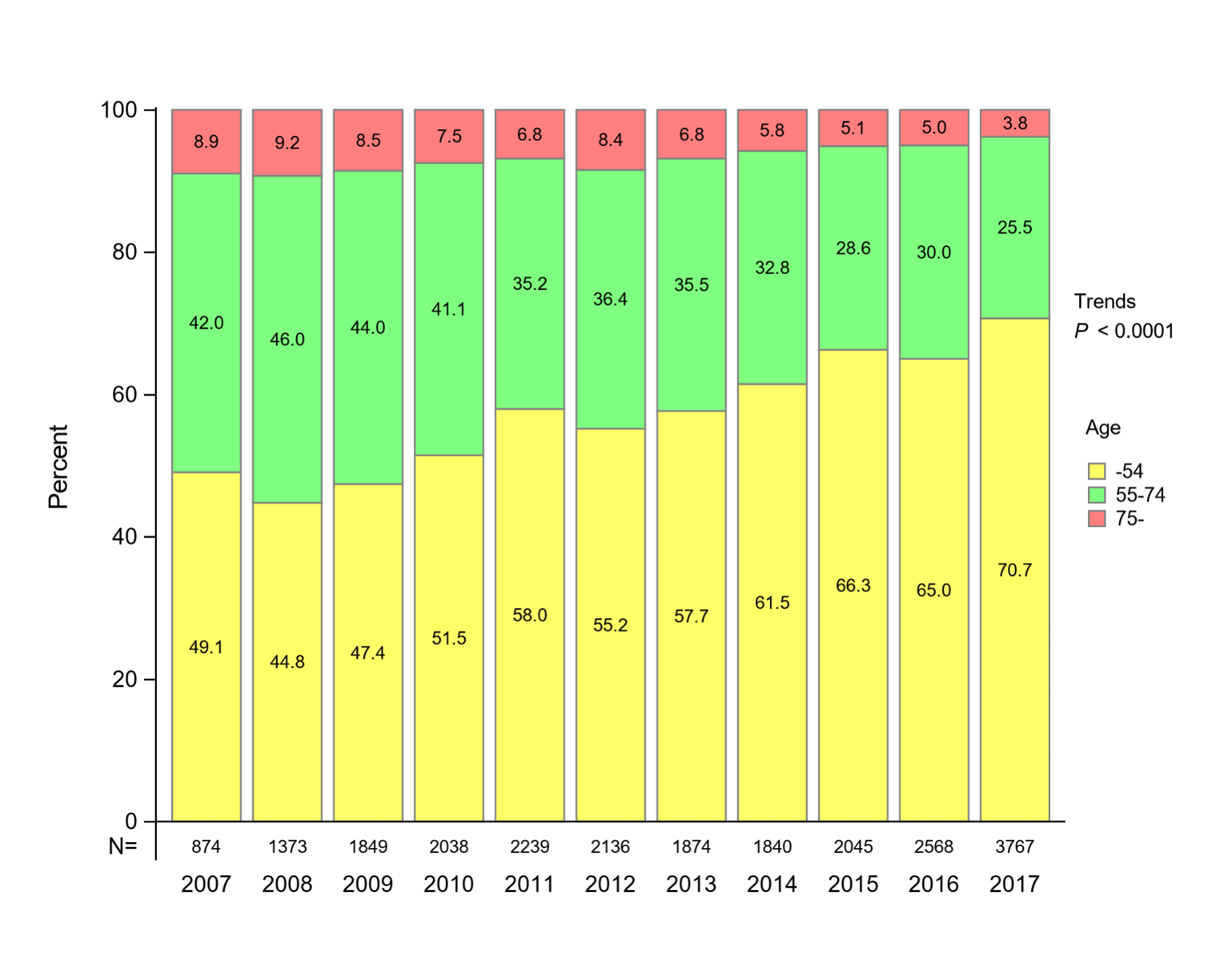
 The trend was analyzed with Mantel-Haenszel statistics. Data were retrieved from GynOp and the National Patient Register. Reference: http://www.gynop.se/home/. Accessed 16 July 2022.

Figure S1B. Distribution of age class in the background female population


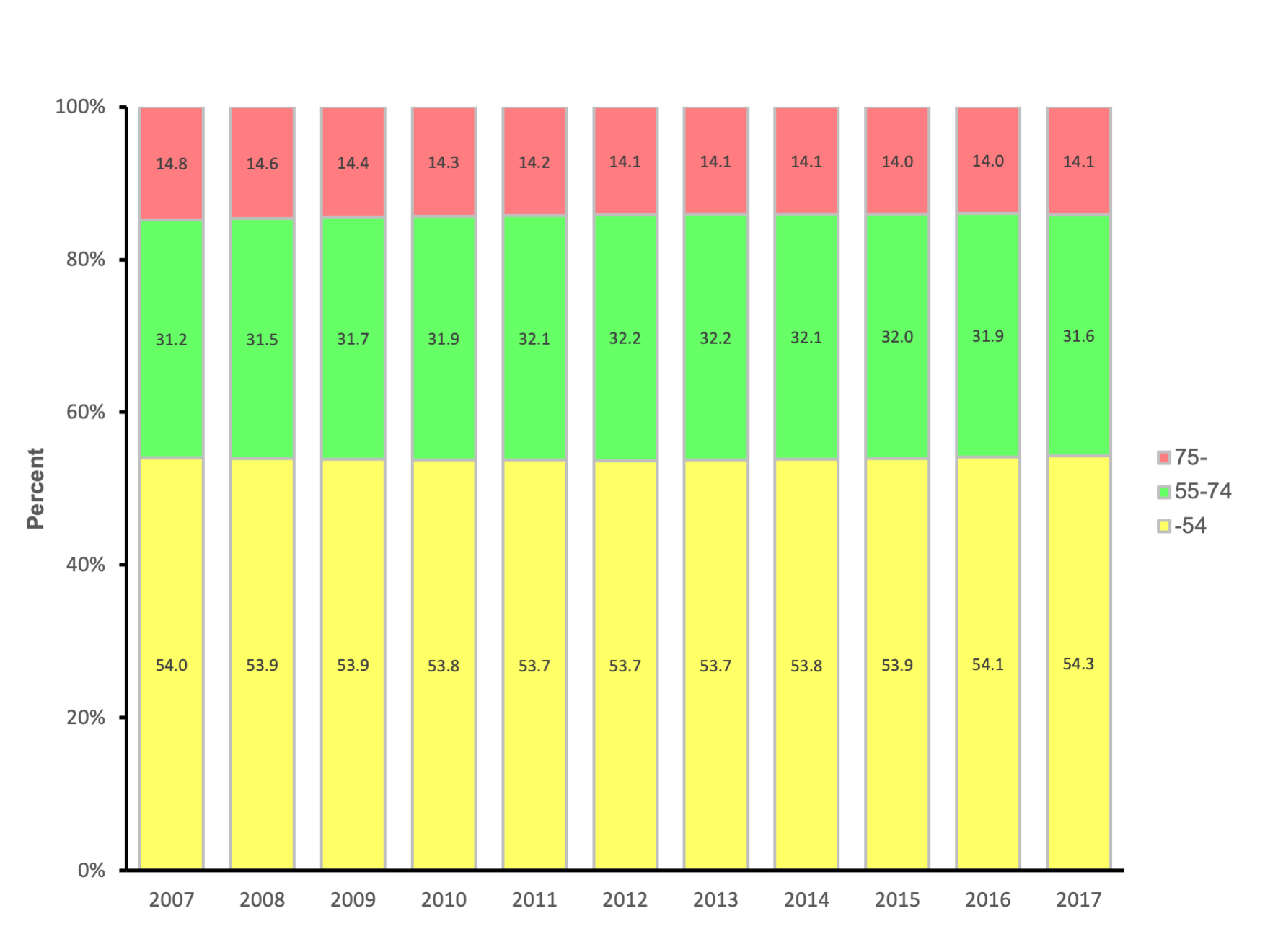


Data retrieved from the Total Population Register by Statistics Sweden (<https://www.scb.se/en/finding-statistics/statistics-by-subject-area/population/population-composition/population-statistics/>). Accessed 16 July 2022.

Figure S2 A+B. Change in frequency of leakage, satisfaction, and improvement postoperatively.

A. Age group 55-74 years


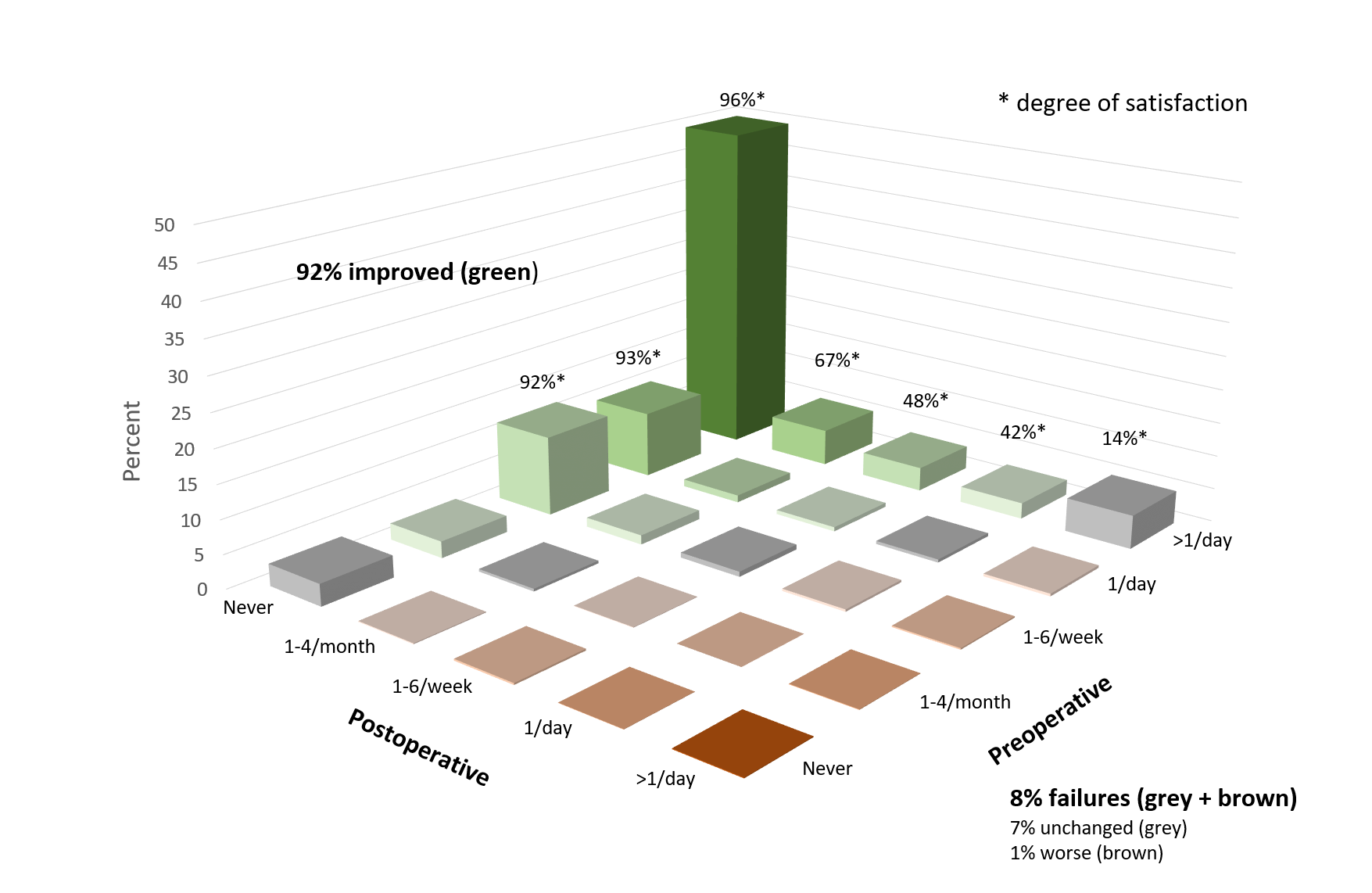


B. Age group ≥75years


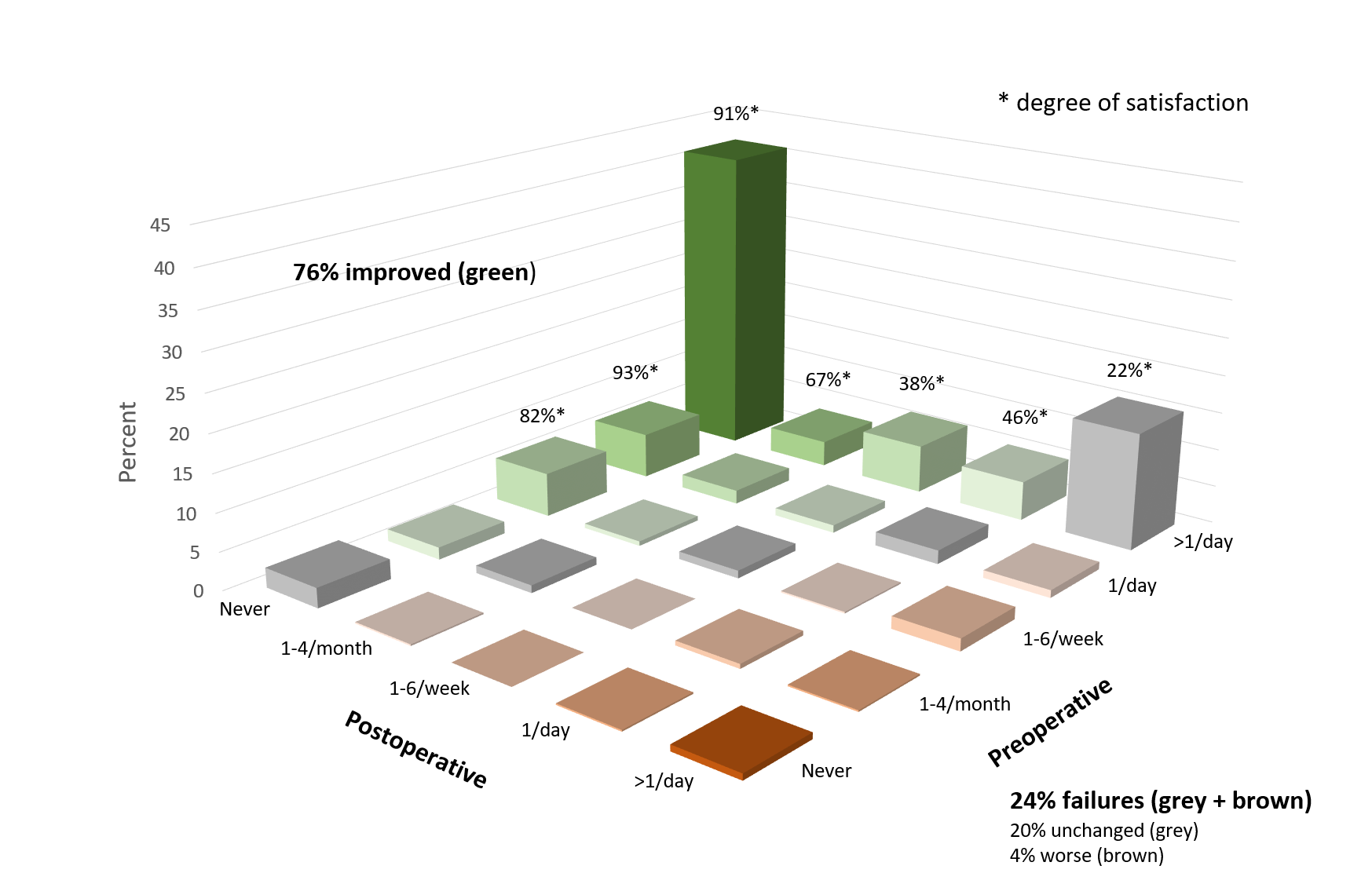


Crude data for the figure are shown in Table S1 A+B and Table S2 A+B.

Figure S3. Rate of failure according to the preoperative rate of leakage


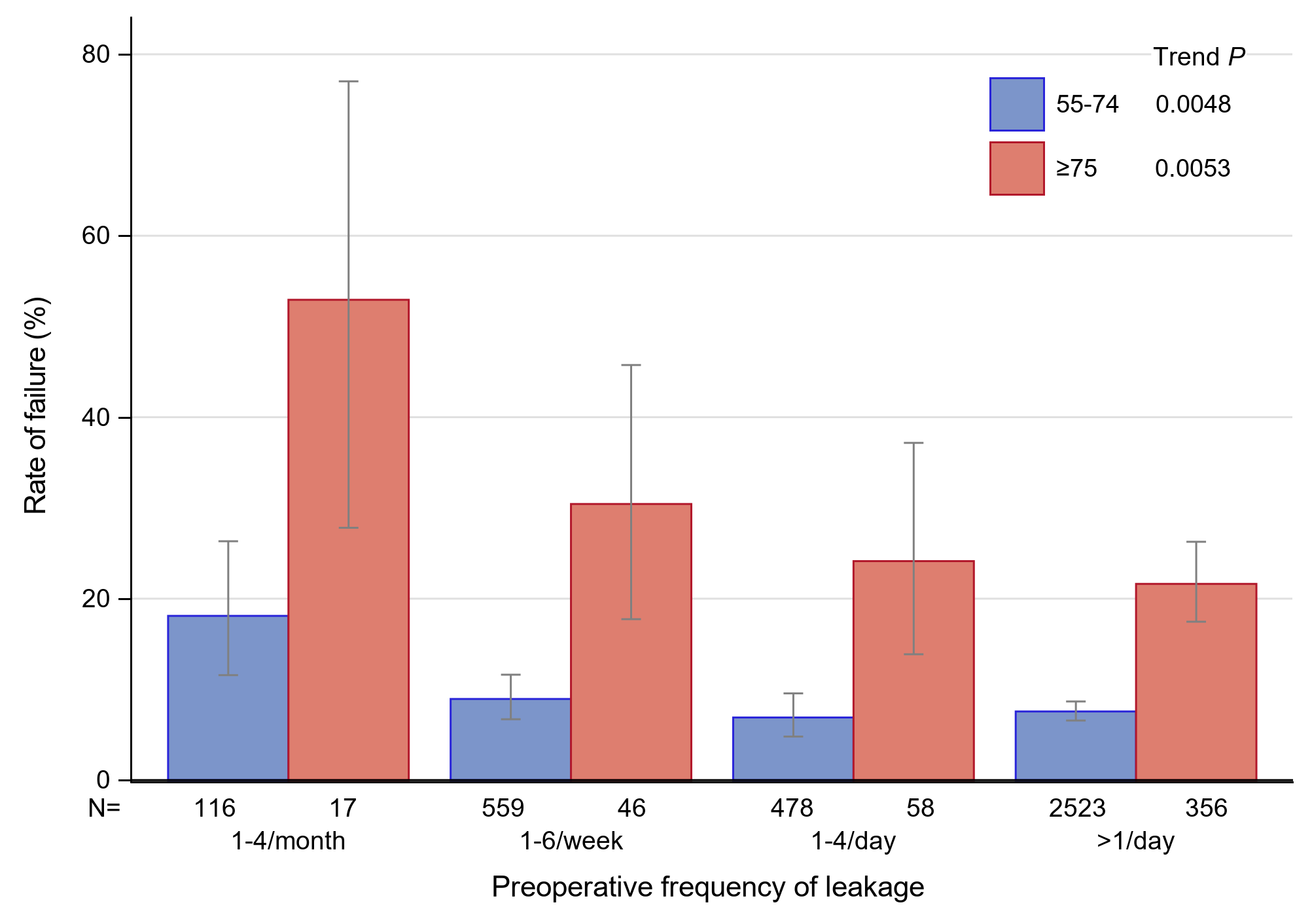


Figure S4. The association between cure and satisfaction according to ASA-class and prior surgery


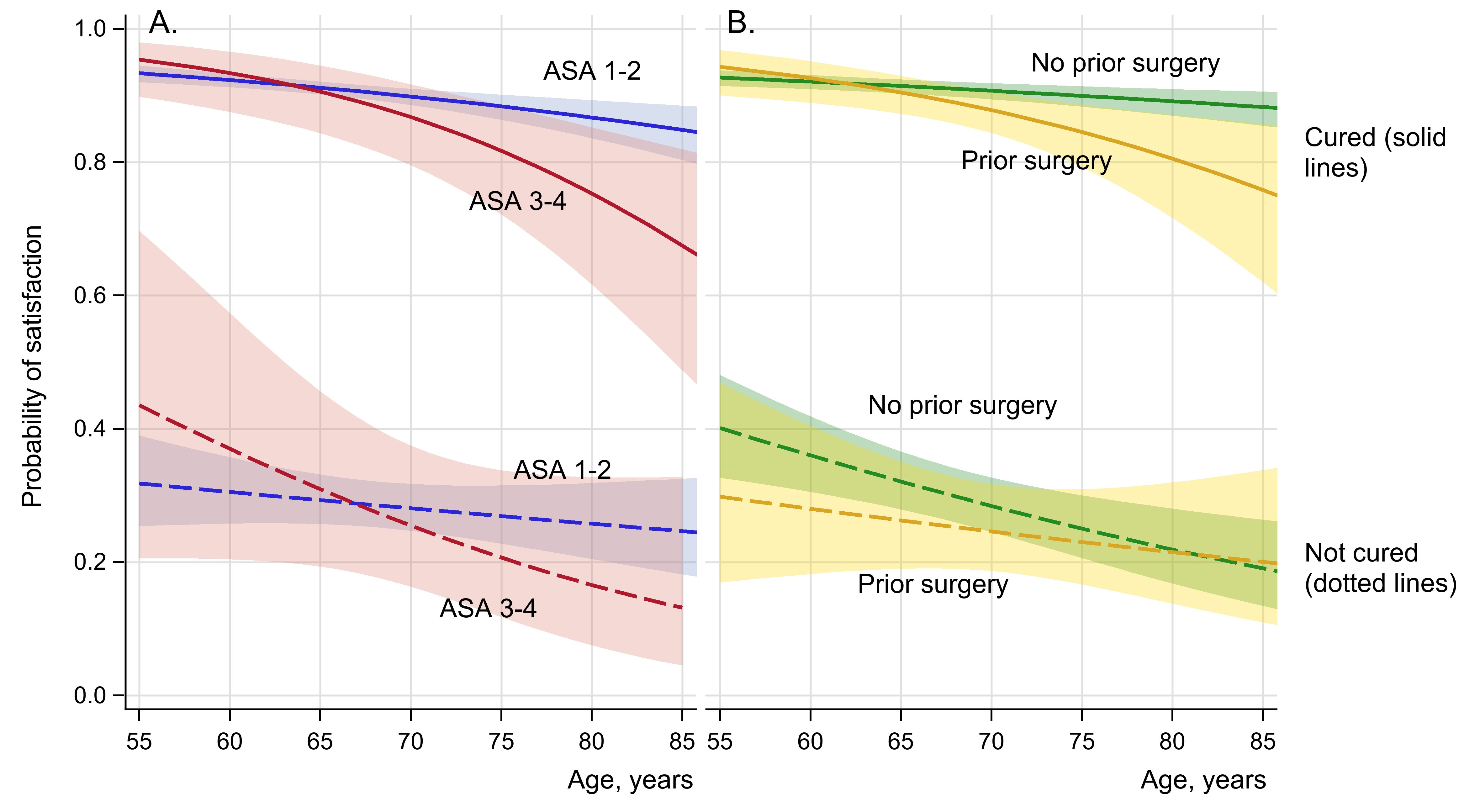


ASA, the classification system adopted by the American Society of Anesthesiologists. The estimated age-related probability (0-1) of satisfaction was calculated from logistic regression models, one for each group (cured and not cured) and ASA classes. The shaded areas show the 95%, 2-tailed confidence interval for the estimated probability of the mean. Prior incontinence surgery as reported in the questionnaire.

Table S1. Change in frequency of leakage

A.

| Women 55–74 years  n/N 3822/4534 (84.3%) | | | | | | |
| --- | --- | --- | --- | --- | --- | --- |
| Frequency of leakage 1-year postoperatively | | | | | | Frequency  of leakage preoperatively |
| n (%)  (95% CI)^a^ | | | | | |  |
| Total | Never | 1-4/month | 1-6/week | 1/day | >1/day |  |
| 2523 (66.0)  (64.5-67.4) | 1894 (49.6)  (46.8-50.0) | 210 (5.5)  (4.8-6.3) | 137 (3.6)  (3.0-4.2) | 91 (2.4)  (1.9-2.9) | 191 (5.0)  (4.4-5.7) | >1/day |
| 478 (12.5)  (11.5-13.6) | 380 (9.9)  (9.0-10.9) | 41 (1.1)  (0.8-1.5) | 24 (0.6)  (0.4-1.0) | 18 (0.5)  (0.3-0.7) | 15 (0.4)  (0.2-0.6) | 1/day |
| 559 (14.6)  (13.6-15.8) | 456 (11.9)  (10.9-13.0) | 53 (1.4)  (1.1-1.8) | 29 (0.8)  (0.5-1.9) | 13 (0.3)  (0.2-0.6) | 8 (0.2)  (0.1-0.4) | 1-6/week |
| 116 (3.0)  (2.5-3.6) | 95 (2.5)  (2.0-3.0) | 15 (0.4)  (0.2-0.6) | 3 (0.1)  (0.0-0.2) | 0 (0.0)  (0.0-0.0) | 3 (0.1)  (0.0-0.2) | 1-4/month |
| 146 (3.8)  (3.3-4.5) | 125 (3.3)  (2.8-3.9) | 5 (0.1)  (0.1-0.3) | 10 (0.3)  (0.1-0.5) | 2 (0.1)  (0.0-0.2) | 4 (0.1)  (0.0-0.3) | Never^b^ |
| 3822 (100.0) | 2950 (77.2)  (75.8-78.5) | 324 (8.5)  (7.6-9.4) | 203 (5.3)  (4.6-6.1) | 124 (3.2)  (2.7-3.9) | 221 (5.8)  (5.1-6.6) | Total |

B.

| Women ≥75years  n/N 496/666 (74.5%) | | | | | | |
| --- | --- | --- | --- | --- | --- | --- |
| Frequency of leakage 1-year postoperatively | | | | | | Frequency  of leakage preoperatively |
| n (%)  (95% CI)^a^ | | | | | |  |
| Total | Never | 1-4/month | 1-6/week | 1/day | >1/day |  |
| 356 (71.8)  (67.7-75.6) | 204 (41.1)  (36.9-45.5) | 17 (3.4)  (2.2-5.4) | 32 (6.5)  (4.6-9.0) | 26 (5.2)  (3.6-7.6) | 77 (15.5)  (12.6-19.0) | >1/day |
| 58 (11.7)  (9.2-14.8) | 30 (6.1)  (4.3-8.5) | 9 (1.8)  (1.0-3.4) | 5 (1.0)  (0.4-2.3) | 9 (1.8)  (1.0-3.4) | 5 (1.0)  (0.4-2.3) | 1/day |
| 46 (9.3)  (7.0-12.1) | 29 (5.9)  (4.1-8.3) | 3 (0.6)  (0.2-1.8) | 5 (1.0)  (0.4-2.3) | 1 (0.2)  (0.0-1.1) | 8 (1.6)  (0.8-3.2) | 1-6/week |
| 17 (3.4)  (2.2-5.4) | 8 (1.6)  (0.8-3.2) | 5 (1.0)  (0.4-2.3) | 0 (0.0)  (0.0-0.0) | 3 (0.6)  (0.2-1.8) | 1 (0.2)  (0.0-1.1) | 1-4/month |
| 19 (3.8)  (2.5-5.9) | 13 (2.6)  (1.5-4.4) | 1 (0.2)  (0.0-1.1) | 0 (0.0)  (0.0-0.0) | 1 (0.2)  (0.0-1.1) | 4 (0.8)  (0.3-2.1) | Never^b^ |
| 496 (100.0) | 284 (57.3)  (52.9-61.5) | 35 (7.1)  (5.1-9.7) | 42 (8.5)  (6.3-11.3) | 40 (8.1)  (6.0-11.8) | 95 (19.2)  (15.9-22.8) | Total |

CI, confidence interval, ^a^The calculation of the 95% confidence limits was based on the exact method. ^b^The answers “Never” preoperatively were not excluded. The tables describe the 25possible pairs of the pre-compared with the postoperative frequency of leakage. The sum of the 25 pairs adds up to 100.0% (3822 women aged 55-74 years, panel A; 496 women ≥75 years old, panel B).

Table S2. The rate of outcome measures versus the preoperative frequency of leakage

A.

| Women 55–74 years  n=3822 | | | | | | |
| --- | --- | --- | --- | --- | --- | --- |
| Preoperative  frequency | All^a^  n = 3676  (100.0%) | Better  n = 3381  (92.0%) | Unchanged  n = 253  (6.9%) | Worse  n = 42  (1.1%) | Failure  n = 295  (8.0%) | Trend^b^ |
|  | n (%)  (95%CI)^3^ | | | | | *P*_1_ value  *P*_2_ value |
| 1-4/month | 116 | 95 (81.9)  (73.7-88.4) | 15 (12.9)  (7.4-20.4) | 6 (5.2)  (1.9-10.9) | 21 (18.1)  (11.6-26.3) | 0.0048  <0.0001 |
| 1-6/week | 559 | 509 (91.1)  (88.4-93.3) | 29 (5.2)  (3.5-7.4) | 21 (3.8)  (2.3-5.5) | 50 (8.9)  (6.7-11.6) |  |
| 1/day | 478 | 445 (93.1)  (90.4-95.2) | 18 (3.8)  (2.3-5.9­) | 15 (3.1)  (1.8-5.1) | 33 (6.9)  (4.8-9.6) |  |
| >1/day | 2523 | 2332 (92.4)  (91.3-93.4) | 191 (7.6)  (6.6-8.7) | 0 (0.0)  (-) | 191 (7.6)  (6.6-8.7) |  |

B.

| Women ≥75 years  n=496 | | | | | | |
| --- | --- | --- | --- | --- | --- | --- |
| Pre-operative frequency | All^a^  n=477  (100.0%) | Better  n=363  (76.1%) | Unchanged  n=96  (20.1%) | Worse  n=18  (3.8%) | *Failure  n=114  (23.9%) | Trend^b^ |
|  | n (%)  (95%CI)^3^ | | | | | *P*_1_ value  *P*_2_ value |
| 1-4/month | 17 | 8 (47.1)  (23.0-72.2) | 5 (29.4)  (10.3-56.0) | 4 (23.5)  (6.8-49.9) | 9 (52.9)  (27.8-77.0) | 0.0053  <0.0001 |
| 1-6/week | 46 | 32 (69.6)  (54.3-82.3) | 5 (10.9)  (3.6-23.6) | 9 (19.6)  (9.4-33.9) | 14 (30.4) (17.7-45.8) |  |
| 1/day | 58 | 44 (75.9)  (62.8-86.1) | 9 (15.5)  (7.4-27.4) | 5 (8.6)  (2.9-19.0) | 14 (24.1)  (13.9-37.2) |  |
| >1/day | 356 | 279 (78.4)  (73.7-82.5) | 77 (21.6)  (17.5-26.3) | 0 (0.0)  (0-0) | 77 (21.6)  (17.5-26.3) |  |

*“Failure” is the sum of “Unchanged” and “Worse”, i.e., the same or a higher frequency of leakage postoperatively. ^a^Women were excluded if they answered “Never” on the question about SUI leakage preoperatively [n=146 (aged 55-74), n=19 (aged ≥75 years)] on the ground that the answer was unreasonable. ^b^The analysis of trend was performed on ordered categories of the independent and the dependent variables. Mantel-Haenszel statistics were used to analyse the trend in rates of the 4 categories of the outcome. ^3^The calculation of the 95% CI of the mean for categorical variables was based on the exact method. The first *p* value (*P*_1_) refers to the analysis of “Better” versus “Failure” and the second *p* value (*P*_2_) analysed the trend between “Better”, “Unchanged”, and “Worse”. The difference of “Failure” between the age groups was significant (*p*<0.0001).

Table S3. Outcomes 12 months postoperatively according to ASA-class

|  | ASA 1-2 | ASA 3-4 | Difference  between cohorts^a^ |
| --- | --- | --- | --- |
|  | N = 4881  % (95%CI) | N = 247  % (95%CI) | *p* value |
| Cure^b^ SUI | 3502/4185  83.7 (82.5-84.8) | 131/200  65.5 (58.5-72.1) | < 0.0001 |
| Cure^b^ UI | 3114/4290  72.6 (71.2-73.9) | 111/207  53.6 (46.6-60.6) | < 0.0001 |
| Satisfaction | 3407/4227  80.6 (79.4-81.8) | 132/201  65.7 (58.7-72.2) | < 0.0001 |
| Improvement | 3680/4211  87.4 (86.4-88.4) | 148/203  72.9 (66.2-78.9) | < 0.0001 |
| *De Novo* | | | |
| Urgency ≥1/week | 236/1707  13.8 (12.2-15.6) | 17/54  31.5 (19.5-45.6) | 0.0018 |
| Nocturia ≥2/night | 209/3080  6.8 (5.9-7.7) | 12/106  11.3 (6.0-18.9) | 0.12 |
| Difficulty emptying the bladder ≥1/week | 387/3443  11.2 (10.2-12.3) | 22/138  15.9 (10.3-23.1) | 0.13 |
| Remission | | | |
| Urgency <1/week | 1272/2402  53.0 (50.9-55.0) | 64/141  45.4 (37.0-54.0) | 0.097 |
| Nocturia <2/night | 486/1167  41.6 (38.8-44.5) | 32/94  34.0 (24.6-44.5) | 0.18 |
| Difficulty emptying the bladder <1/week | 369/603  61.2 (57.2-65.1) | 26/50  52.0 (37.4-66.3) | 0.26 |
| Complications | | | |
| Bladder perforation | 229/4457  5.1 (4.5-5.8) | 19/220  8.6 (5.3-13.2) | 0.046 |
| Ureteric injury | 9/4457  0.2 (0.1-0.4) | 2/220  0.9 (0.1-3.3) | 0.18 |
| Fistulae | 4/4457  0.1 (0.0-0.2) | 1/220  0.5 (0.0-2.5) | 0.43 |
| Readmission within 30 days | 54/4881  1.1 (0.8-1.4) | 1/247  0.4 (0.0-2.2) | 0.50 |
| Mortality 30 within days | 1/4881  0.0 (0.0-0.1) | 0/247  0.0 (0.0-.) | 1.00 |

ASA; American Society of Anesthesiologists classification of general health; UI; urinary incontinence, SUI; stress UI. Prior surgery denotes incontinence procedures only as reported in the questionnaire. ^a^For comparison between groups, Fisher´s Exact test (lowest 1-sided *P* value multiplied by 2) was used for dichotomous variables. ^b^Cure was defined as incontinence “Never” and “1-4 times per month”.

Table S4. Outcomes 12 months postoperatively grouped according to prior surgery for stress urinary incontinence

|  | No prior UI surgery  N = 3798 | Prior^a^ UI surgery  N = 801 | Difference  between cohorts^b^ |
| --- | --- | --- | --- |
|  | n/N  % (95%CI) | n/N  % (95%CI) | *p* value |
| Satisfaction | 2674/3282  81.5 (80.1-82.8) | 488/685  71.2 (67.7-74.6) | < 0.0001 |
| Improvement | 2876/3261  88.2 (87.0-89.3) | 545/686  79.4 (76.2-82.4) | < 0.0001 |
| Cured^c^ (SUI) | 2744/3254  84.3 (83.0-85.6) | 493/671  73.5 (70.0-76.8) | < 0.0001 |
| Cured^c^ (UI) | 2454/3326  73.8 (72.3-75.3) | 425/702  60.5 (56.8-64.2) | < 0.0001 |
| *De Novo* | | | |
| Urgency | 175/1342  13.0 (11.3-15.0) | 50/209  23.9 (18.3-30.3) | < 0.0001 |
| Nocturia ≥ 2/night | 165/2414  6.8 (5.9-7.9) | 33/424  7.8 (5.4-10.8) | 0.54 |
| Difficulty emptying the bladder <1/week | 312/2698  11.6 (10.4-12.8) | 53/497  10.7 (8.1-13.7) | 0.62 |
| Complications | | | |
| Bladder injury | 169/3459  4.9 (4.2-5.7) | 46/728  6.3 (4.7-8.3) | 0.14 |
| Ureteric injury | 6/3459  0.2 (0.1-0.4) | 2/728  0.3 (0.0-1.0) | 0.84 |
| Fistula | 0/3459  0.0 (0.0-.) | 2/728  0.3 (0.0-1.0) | 0.060 |

CI denotes confidence interval. Non-responders: no prior surgery 12.0% (455/3798); prior surgery 12.0% (96/801). ^a^ Prior surgery denotes incontinence procedures only as reported in the questionnaire. ^b^ Fisher´s Exact test (lowest 1-sided *p value* multiplied by 2) was used to compare groups for dichotomous variables. ^c^ Cure was defined as incontinence “Never” and “1-4 times per month”.
